# Modified arteriosclerosis score predicts the outcomes of diabetic kidney disease

**DOI:** 10.1186/s12882-021-02492-x

**Published:** 2021-08-18

**Authors:** Yifan Zhang, Qifeng Jiang, Jianteng Xie, Chunfang Qi, Sheng Li, Yanhui Wang, Yau Hok Him, Zujiao Chen, Shaogui Zhang, Qiuling Li, Yuan Zhu, Ruizhao Li, Xinling Liang, Xiaoyan Bai, Wenjian Wang

**Affiliations:** 1 The Second School of Clinical Medicine, Southern Medical University, Guangzhou, 510515 China; 2 Division of Nephrology, Guangdong Provincial People’s Hospital, Guangdong Academy of Medical Sciences, 106 Zhongshan Er Road, Main Building, Room 1436, Guangzhou, 510080 Guangdong China; 3 Division of Nephrology, Wenzhou Central Hospital, Wenzhou, 325000 China; 4 Division of Renal Pathology, Guangzhou KingMed Diagnostic Laboratory LTD, Guangzhou, 510320 China; 5 School of Medicine, South China University of Technology, Guangzhou, 510006 China; 6 Division of Nephrology, The First Affiliated Hospital of Wenzhou Medical University, Wenzhou, 325000 China; 7 Shantou University Medical College, Shantou, 515041 China; 8 Division of Nephrology, Wenzhou People’s Hospital, Wenzhou, 325000 China

**Keywords:** Arteriosclerosis, Diabetic kidney disease (DKD), Nomogram, Outcomes, Renal pathology

## Abstract

**Background:**

The significance of renal arteriosclerosis in the prediction of the renal outcomes of diabetic kidney disease (DKD) remains undetermined.

**Methods:**

We enrolled 174 patients with DKD from three centres from January 2010 to July 2017. The severity and extent of arteriosclerosis were analysed on sections based on dual immunohistochemical staining of CD31 and α-smooth muscle actin. An X-tile plot was used to determine the optimal cut-off value. The primary endpoint was renal survival (RS), defined as the duration from renal biopsy to end-stage renal disease or death.

**Results:**

The baseline estimated glomerular filtration rate (eGFR) of 135 qualified patients was 45 (29 ~ 70) ml/min per 1.73 m^2^, and the average 24-h urine protein was 4.52 (2.45 ~ 7.66) g/24 h. The number of glomeruli in the biopsy specimens was 21.07 ± 9.7. The proportion of severe arteriosclerosis in the kidney positively correlated with the Renal Pathology Society glomerular classification (*r* = 0.28, *P* < 0.012), interstitial fibrosis and tubular atrophy (IFTA) (*r* = 0.39, *P <* 0.001), urine protein (*r* = 0.213, *P* = 0.013), systolic BP (*r* = 0.305, *P* = 0.000), and age (*r* = 0.220, *P* = 0.010) and significantly negatively correlated with baseline eGFR (*r* = − 0.285, *P* = 0.001). In the multivariable model, the primary outcomes were significantly correlated with glomerular class (HR: 1.72, CI: 1.15 ~ 2.57), IFTA (HR: 1.96, CI: 1.26 ~ 3.06) and the modified arteriosclerosis score (HR: 2.21, CI: 1.18 ~ 4.13). After risk adjustment, RS was independently associated with the baseline eGFR (HR: 0.97, CI: 0.96 ~ 0.98), urine proteinuria (HR: 1.10, CI: 1.04 ~ 1.17) and the modified arteriosclerosis score (HR: 2.01, CI: 1.10 ~ 3.67), and the nomogram exhibited good calibration and acceptable discrimination (C-index = 0.82, CI: 0.75 ~ 0.87).

**Conclusions:**

The severity and proportion of arteriosclerosis may be helpful prognostic indicators for DKD.

**Supplementary Information:**

The online version contains supplementary material available at 10.1186/s12882-021-02492-x.

## Introduction

Diabetic kidney disease (DKD) is the most common complication in patients exposed to long-term hyperglycaemia [1]. In China, the prevalence of DKD is rising dramatically. Diabetes-related chronic kidney disease (CKD) has overtaken glomerulonephritis as the leading cause of end-stage renal disease (ESRD) [2]. Studies have shown that patients with diabetes in Asia are younger than those in Western countries and are more likely to develop ESRD [3, 4]. However, there are still few biomarkers to predict the progression of DKD to ESRD [5]. Although clinical risk factors, including albuminuria and estimated glomerular filtration rate (eGFR) [6], are most commonly used to predict the progression to ESRD, there is still a lack of pathologic markers, such as those associated with the Oxford classification of IgA nephropathy [7–11], partly because the proportion of patients who receive kidney biopsies remains drastically lower than that of patients with primary glomerulonephritis.

In 2010, the Research Committee of the Renal Pathology Society (RPS) developed a pathologic classification system of diabetic nephropathy [12]. However, the connections of pathological lesions with renal prognosis have not been fully established. An evidence-based approach is needed to better define pathologic lesions within the classification of the DKD spectrum, including the significance of vascular damage. Some studies have suggested that Kimmelstiel-Wilson nodules have a higher risk of ESRD than diffuse DKD [13], while others have found segmental sclerosis and extracapillary hypercellularity to be poor prognostic indicators for ESRD [14, 15]. Thus, the prognostic significance of pathological parameters in DKD remains controversial [16].

DKD is a common vascular complication of diabetes. Renal ischaemia and endothelial injury play important roles in the pathological processes that promote renal fibrosis and CKD [17–19]. Although the DKD pathologic classification of RPS takes into account and defines renal vascular damage, its prognostic value has not yet been proven [14, 15]. Because the original arteriosclerosis score for DKD classification was based on the worst artery, this classification only considered the severity of the injured artery but not the proportion of the damaged arteries of all counted arteries.

In the current study, we hypothesized that the proportion of damaged arteries to all arteries in the tissue may contribute to the prognosis of DKD. To validate the significance of renal artery damage in the prediction of renal outcomes of DKD, we employed dual immunoperoxidase staining to better identify arteries with or without lesions and developed a modified arteriosclerosis score for the severity and proportion of all counted arteries. Nomograms were then used to predict the risks of DKD with all clinical characteristics as well as the pathological score [20–22].

## Methods

### Study design and population

This was a multi-centre, retrospective study of patients with DKD diagnosed by renal pathology. Patients were enrolled between January 2010 and July 2017 from three hospitals (Guangdong Provincial People’s Hospital, Wenzhou Central Hospital and Wenzhou People’s Hospital). Of the 174 patient-included cohorts, 135 patients were eventually enrolled. The exclusion criteria included patients with DKD coincident with other patterns of kidney injury and inadequate renal tissue samples (renal tissue section should contain at least 10 glomeruli) [12].

All demographic and clinical data were carefully documented from electronic medical clinical records of three hospitals. Demographics included age at the time of biopsy and sex. Clinical parameters collected included body mass index (BMI), blood pressure, hypertension, diabetic retinopathy (DR), diabetes duration, smoking, glycosylated haemoglobin (HbA1c) level, total cholesterol (Chol) level, serum albumin (ALB) level, urine protein (UPRO) level, eGFR, follow-up time, and follow-up serum creatinine (sCr) level. Hypertension was considered in patients with systolic blood pressure greater than 140 mmHg, diastolic blood pressure greater than 90 mmHg or antihypertensive drug administration. DR was defined as the presence of any characteristic lesion as described by the International Clinical Diabetic Retinopathy Disease Severity Scale, which is a grading standard designed according to the Wisconsin Epidemiologic Study of Diabetic Retinopathy (WESDR). Hyperuricaemia was defined as serum uric acid ≥420 μmol/L in males or ≥ 360 μmol/L in females. Urinary protein was expressed in g per 24 h. The eGFR was estimated using the creatinine-based Chronic Kidney Disease Epidemiology Collaboration equation [23].

### Outcomes

The clinical outcomes were considered to evaluate the predictive value of renal pathology variables, ESRD (i.e., haemodialysis, peritoneal dialysis for ESRD, or kidney transplantation), or all-cause death. The follow-up time was defined as the duration from renal biopsy to ESRD or all-cause death [24, 25], the date of last patient contact, or the study end date of July 2017.

### Pathology evaluation

All renal biopsies were performed and processed by standard techniques [24, 25]. The diagnosis of DKD was completed with the findings of light microscopy, immunofluorescence microscopy and electron microscopy as well as supportive clinical information. Any coexisting disorders were reviewed to rule out other accompanying glomerular diseases. In addition, dual immunohistochemistry of the paraffin sections with an antibody against α-smooth muscle actin (α-SMA, NCL-L-SMA, Aq-meditech, China) and an antibody against CD31 (NCL-L-CD31–607, Aq-meditech, China), which is an endothelial marker, was performed to highlight the arteries.

Glomerular lesions and interstitial lesions were scored according to the DKD pathologic classification issued by RPS [24, 25]. For vascular lesions of DKD, a modified arteriosclerosis score and modified arteriolar hyalinosis score [26] were employed on dual immunoperoxidase staining sections, which could be more convenient to distinguish arterial damage. In addition, the thickness of the basement membrane under electron microscopy was added as one of the pathological characteristics.

To assess the proportion of all involved arteries distributed in the tissues, severe arteriosclerosis, which was defined as arteries with thickening exceeding the intima thickness, to all arteries in sections was calculated. Since the proportion of severe arteriosclerosis was a continuous variable, X-tile software was used to find the best cut-off value and convert it to hierarchical data [27, 28]. Eventually, the optimal cut-off value for the proportion of severe arteriosclerosis was defined as 50%. We scored extent of arteriosclerosis together as a percentage of the total involved arteries of all severe arteriosclerosis. A score of 0 was assigned when the biopsy specimen showed no arteriosclerosis, a score of 1 was assigned when less than 50% severe arteriosclerosis was present, and a score of 2 was assigned when more than 50% severe arteriosclerosis was present. RPS glomerular class, IFTA, interstitial inflammation, arteriolar hyalinosis score, and arteriolar hyalinosis score were determined following standard procedures [24, 25].

Without knowledge of the clinical outcomes, two experienced nephropathologists (Qifeng Jiang and Yifan Zhang) were assigned to independently evaluate and score light microscopy slides. Pathological scoring disagreements were resolved by consensus.

### Statistical analyses

Variables with a normal distribution are presented as the means and standard deviations and were subjected to unpaired *Student’s t*-test between groups, one-way analysis of variance (*ANOVA*) in multiple groups, or *Pearson’s* test for correlation analysis. Nonparametric variables are presented as the medians and interquartile ranges (IQR) and were analysed with the *Mann–Whitney* test between groups, *Kruskal–Wallis* test in multiple groups, or *Spearman* rank correlation test. Categorical variables were expressed as percentages and compared using the *Pearson* χ^2^ test.

The modified arteriosclerosis score cut-off value was analysed using the X-tile plot [28]. Survival analysis for renal survival or death was performed using *Kaplan-Meier* (KM) survival curves with log-rank tests and the *Cox* proportional hazards model to identify the potential association between clinical and pathologic variables and outcomes. Variables with a significance of *P* < 0.1 in the univariate models were included in the multivariable models [29]. Three multivariable models were established, and the first multivariable model (the “Clinical Model”) included only those clinical variables significant to *P* < 0.1 in the univariate models. The second multivariable model (the “Pathological Model”) included only those pathological variables significant to *P* < 0.1 in the univariate models. The “Fully Risk-Adjustment Model” incorporated clinical variables significant at *P* < 0.05 in the clinical model, as well as pathological variables significant at *P* < 0.05 in the pathological model. Then, the AUCs of different risk variables and two models in predicting RS were estimated using time-dependent receiver operating curves (ROCs).

To estimate median and individual postdiagnosis renal survival probabilities at 1, 3, and 5 years, a nomogram was constructed based on the “Fully Risk-Adjustment Model” [20]. Validation of the prediction model was performed using two parameters: discrimination and calibration [30]. Discrimination was measured by the concordance index (C-index), whereas calibration was evaluated using a calibration plot. By convention, a concordance index of less than 0.6 indicates poor discrimination, 0.60–0.75 indicates possibly helpful discrimination, and more than 0.75 indicates outstanding discrimination [30].

All tests used were two-sided, with a *P*-value < 0.05 considered statistically significant. All statistical analyses were performed using X-tile software version 3.6.1 (http://tissuearray.org), GraphPad Prism 7 software (GraphPad Software) or R version 3.6.3 (Foundation for Statistical Computing, Vienna, Austria; http://www.R-project.org/).

## Results

### Clinical characteristics at presentation and during follow-up

**Fig. 1:**
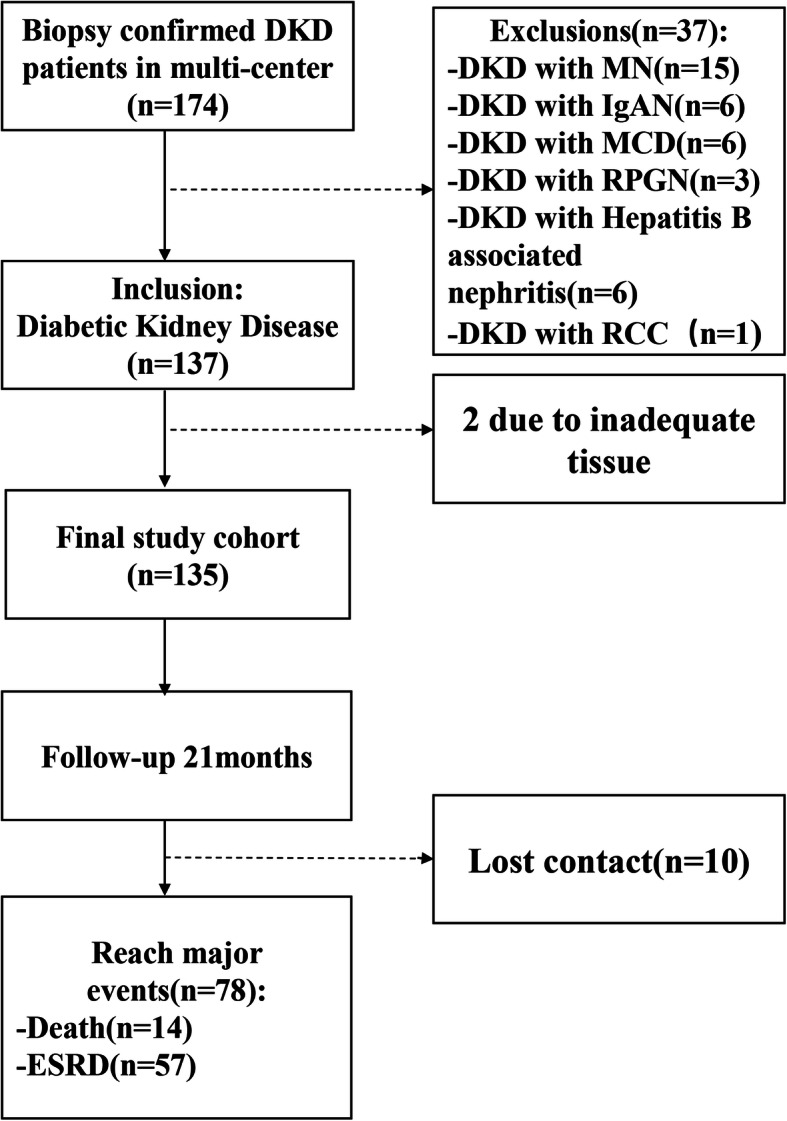
Flow diagram of the enrolment process. Abbreviations: DKD, diabetic kidney disease; ESRD, end-stage renal disease; IgAN, IgA nephropathy; MCD, minimal change disease; MN, membranous nephropathy; RCC, renal cell carcinoma; RPGN, rapidly progressive glomerulonephritis

Of 174 patients with biopsy-proven DKD, 37 patients were excluded due to coincidence with other kidney diseases, and 2 patients were excluded due to inadequate tissue samples. Finally, 135 patients were enrolled in this study. The mean number of glomeruli for each biopsy specimen was 21.07 ± 9.7. During the 21-month follow-up (interquartile range, 15–38 months) [31], 10 patients (7.4%) lost connection, 57 patients (42.2%) developed ESRD, 5 individuals (3.7%) died before reaching ESRD, and 9 individuals (6.7%) died after progression to ESRD. The flowchart of the study is shown in Fig. 1.

The baseline clinical data of 135 DKD patients are listed in Table 1. The average age of the enrolled participants at baseline was 52.13 ± 10.42 years, and the majority of patients were male (71.9%), with a BMI of 24.74 ± 3.88 kg/m^2^. The median duration of diabetes was 7 (3–11) years, with HbA1c of 7.72 ± 3.88%, SBP of 157.12 ± 25.92 mmHg, and total cholesterol of 5.89 (4.9–6.81) mmol/L. The median urine protein at enrolment was 4.52 (2.45–7.66) g/24 h, with a median baseline eGFR of 45 (29–70) ml/min per 1.73 m^2^.

**Table 1:** Clinical characteristics at the time of biopsy in 135 patients with DKD

Characteristics	Results
**Age (years)**	52.13 ± 10.42
**Male sex**	71.9%
**Smoke**	32.6%
**Hypertension**	93.3%
**Hyperuricemia**	42.2%
**Diabetic retinopathy**	65.9%
**Diabetes duration (years)**	7 (3–11)
**BMI (kg/m**^**2**^**)**	24.74 ± 3.88
**Systolic BP (mmHg)**	157.12 ± 25.92
**HbA1c (%)**	7.72 ± 1.78
**Total Cholesterol (mmol/L)**	5.89(4.9–6.81)
**Albumin (g/L)**	27.67 ± 7.13
**Urine protein (g/24 h)**	4.52 (2.45–7.66)
**Baseline eGFR (ml/min per 1.73m**^**2**^**)**	45 (29–70)

Data are expressed as the means±SD, medians (interquartile range), or percentages

*Abbreviations*: *BMI* body mass index, *DKD* diabetic kidney disease, *eGFR* estimated glomerular filtration rate, *HbA1c* glycosylated haemoglobin

The pathological characteristics of the patients are tabulated in Table 2.

**Table 2:** Pathologic scoring of kidney biopsy specimens from 135 patients with DKD

Pathologic scores	Pathologic definitions	Prevalence, n (%)
**RPS Glomerular class**		
I	Mild	1 (0.7%)
IIa	Mild mesangial expansion in > 25% of mesangium	15 (10.2%)
IIb	Severe mesangial expansion in > 25% of mesangium	17 (11.6%)
III	At least one KW lesion	86 (58.5%)
IV	Advanced diabetic glomerulosclerosis	16 (10.9%)
**IFTA**		
0	None	0 (0%)
1	1–25%	25 (18.5%)
2	26–50%	45 (33.3%)
3	> 50	65 (48.2%)
**Interstitial inflammation**		
0	None	0 (0%)
1	Infiltration only in relation to IFTA	88 (65.2%)
2	Infiltration in areas without IFTA	47 (34.8%)
**Arteriolar hyalinosis score**		
0	Absent	3(2.2%)
1	At least one area of arteriolar hyalinosis	12(8.9%)
2	More than one area of arteriolar hyalinosis	120(88.9)
**Modified arteriolar hyalinosis score**		
0	Absent	3(2.2%)
1	Mild-to-moderate PAS-positive hyaline thickening in at least one arteriole	12(8.9%)
2	moderate-to-severe PAS-positive hyaline thickening in more than one arteriole	49(36.3%)
3	Severe PAS-positive hyaline thickening in many arterioles	71(52.6%)
**Arteriosclerosis score**^**a**^		
0	No intimal thickening	0(0%)
1	Intimal thickening less than thickness of media	63(46.7%)
2	Intimal thickening greater than thickness of media	72(53.3%)
**Modified arteriosclerosis score**^**b**^		
1	0–49% The Proportion of arteries with intima thickness greater than media thickness in the biopsy	113(83.7%)
2	≥50%	22(16.3%)

*Abbreviations*: *IFTA* interstitial fibrosis and tubular atrophy, *RPS* the Renal Pathology Society

^a^ original arteriosclerosis score the most severely affected artery in the biopsy; ^b^ Modified arteriosclerosis score the proportion of severe arteriosclerosis in the biopsy

**Fig. 2:**
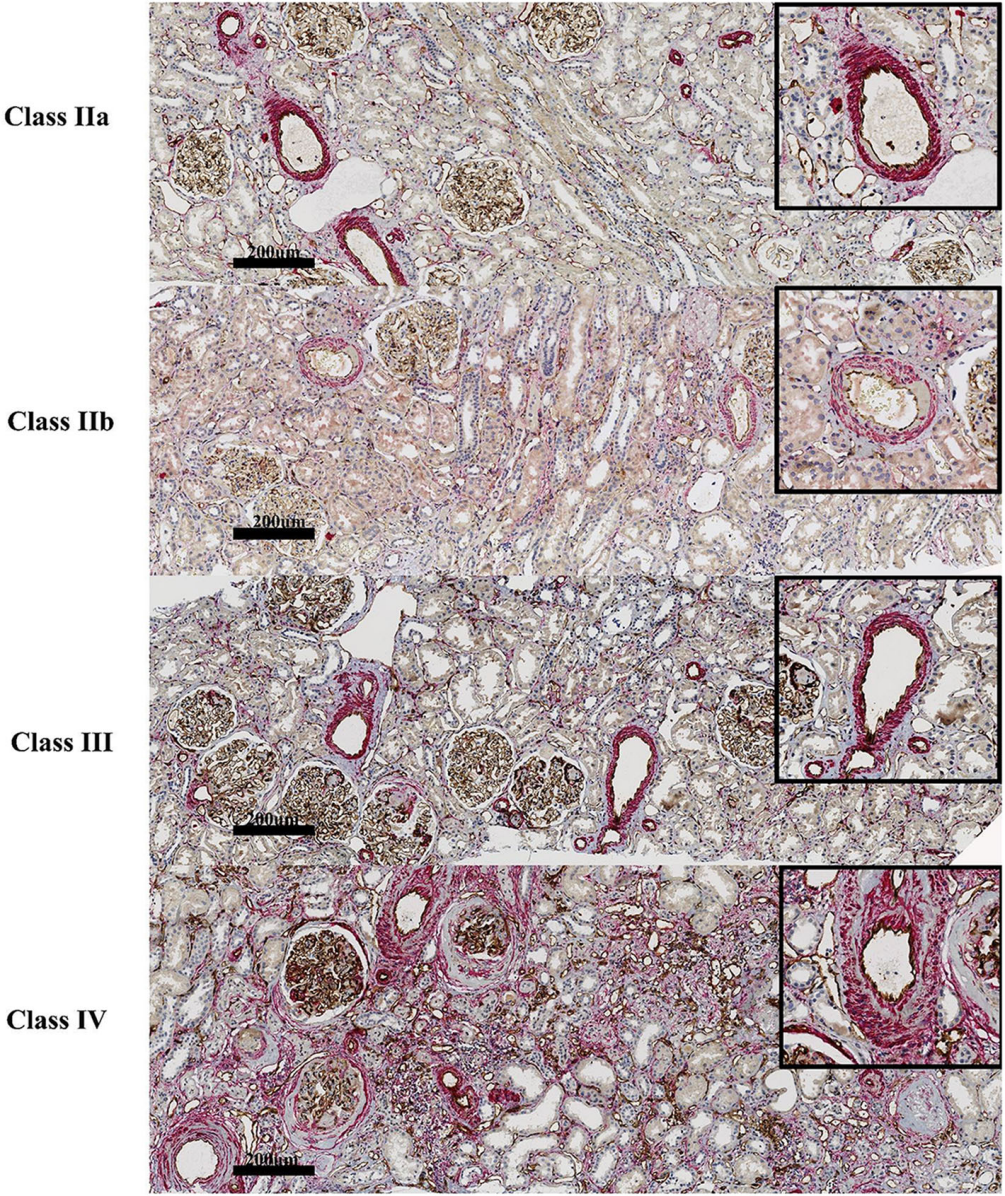
Representative examples of the morphologic lesions in DKD. The four graphs show the morphologic lesions of arteriosclerosis, from mild mesangial expansion (Class IIa) to advanced diabetic glomerulosclerosis (Class IV). Arteriosclerotic lesions are stained with α-CD31 and α-SMA in tissues

**Fig. 3:**
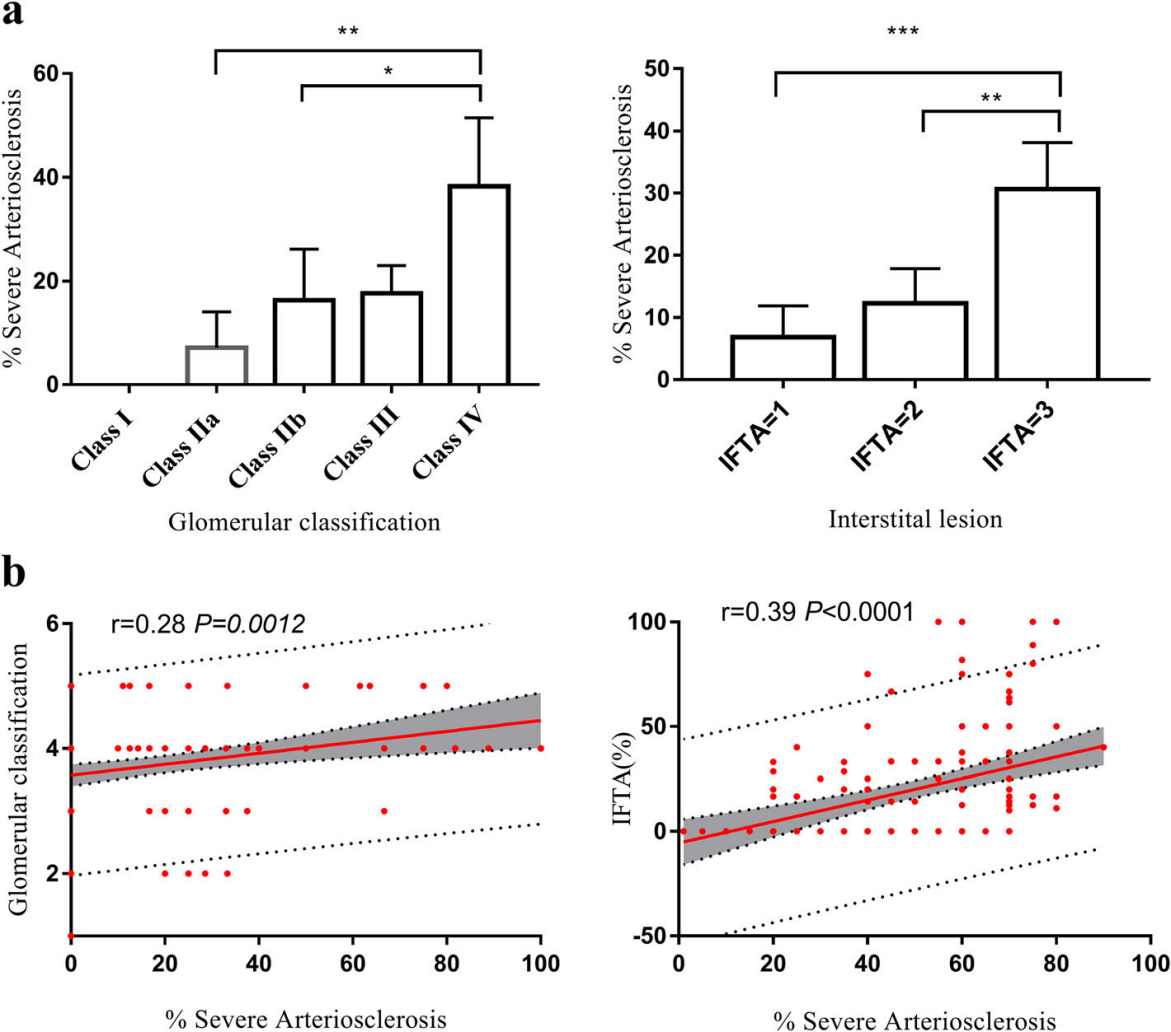
Relationship between vascular damage and glomerular class and IFTA in diabetic nephropathy. **a** Histogram shows the proportion of severe arteriosclerosis changes with glomerular classification and IFTA progression. Figure (**b**) Scatter plot showing the relationship between the proportion of severe arteriosclerosis and glomerular classification and IFTA. Abbreviations: IFTA, interstitial fibrosis and tubular atrophy

As depicted in Fig. 2, the proportion of severe arteriosclerotic lesions exhibited a correlation with glomerular classification (*r* = 0.28, *P*<0.012) and IFTA (*r* = 0.39, *P*<0.001) (Fig. 3a and b). In glomerular class IV, the proportion of severe arteriosclerosis was higher than in other classes, but there was no significant difference between class IIb and class III. In some cases, the severity of arteriosclerosis of class III was not more severe than that of class IIb (Figs. 2 and 3a). IFTA is more closely related to arteriosclerosis, and a continuous increase in progressive fibrosis parallels the increase in the proportion of severe arteriosclerosis (Fig. 3a and b).

**Fig. 4:**
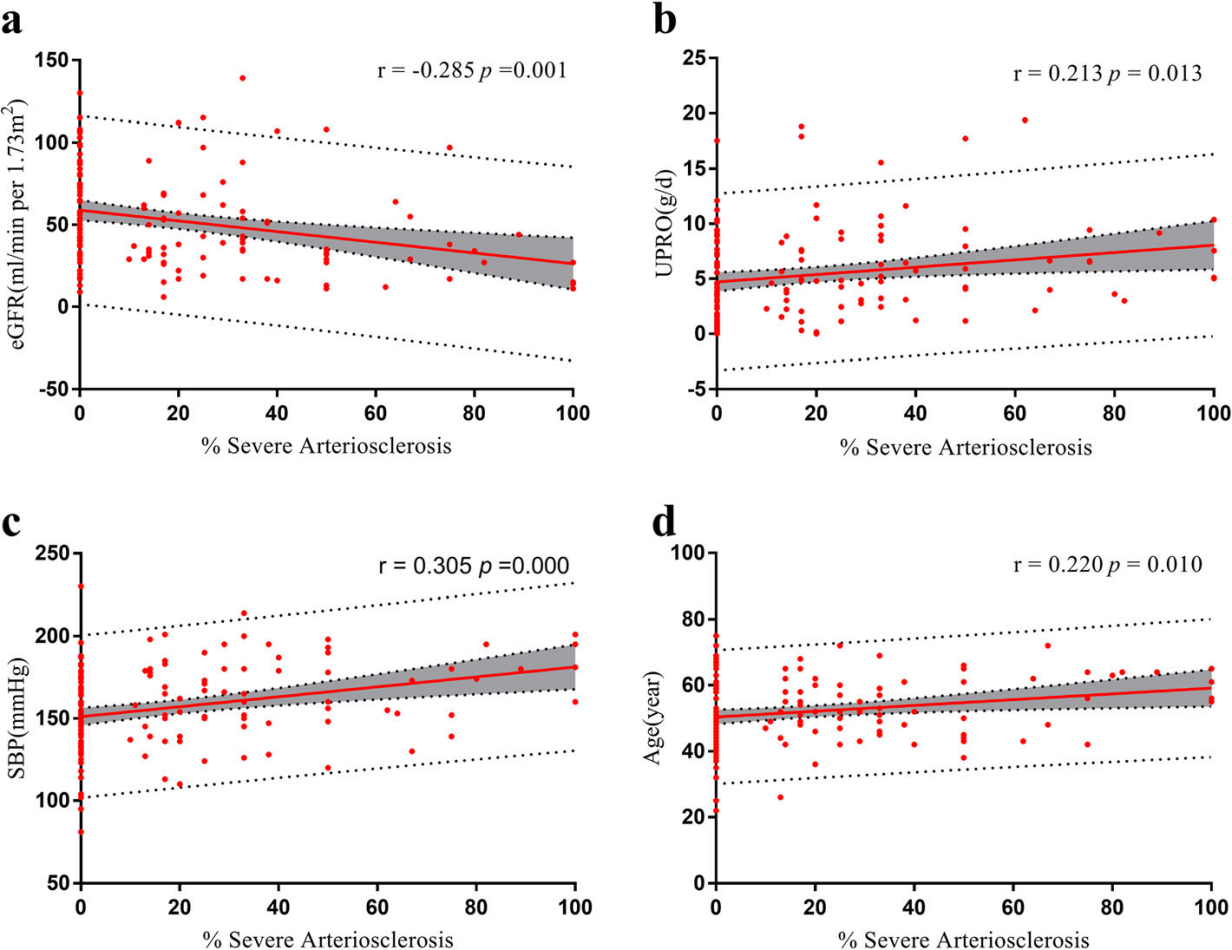
Correlation between the proportion of severe atherosclerosis and clinical variables. **a** The correlation between the proportion of severe atherosclerosis and baseline eGFR. **b** The correlation between the proportion of severe atherosclerosis and UPRO. **c** The correlation between the proportion of severe atherosclerosis and SBP. **d** The correlation between the proportion of severe atherosclerosis and age. All significant correlations are given as Pearson’s r, *P* < 0.05. The grey area represents the 95% confidence interval. Abbreviations: eGFR, estimated glomerular filtration rate; SBP, systolic blood pressure; UPRO, urine protein

Figure 4 shows the correlation between the proportion of severe atherosclerosis and clinical variables. The proportion of severe atherosclerosis was significantly negatively correlated with baseline eGFR (*r* = − 0.285, *P* = 0.001) but significantly positively correlated with urine protein (*r* = 0.213, *P* = 0.013), SBP (*r* = 0.305, *P* = 0.000), and age (*r* = 0.221, *P* = 0.010). Univariable and multivariable Cox regression analyses were used to explore the association between clinical characteristics and renal survival (ESRD or death). Only clinical variables that showed significance in the univariate analysis (*P* < 0.1) were included in the multivariable clinical model. The only clinical variables to remain significant in the multivariable clinical model were baseline eGFR, urine proteinuria and albumin (Table 3).

**Table 3:** Correlations between clinical features and outcome (endpoint:ESRD or death)

Characteristics	Univariate Models HR (95% CI)	***P*** Value	Clinical Model HR (95% CI)	***P*** Value
**Age**	0.99(0.97–1.02)	0.79		
**Female sex**	1.24(0.74–2.07)	0.41		
**Smoke**	0.88(0.50–1.56)	0.66		
**Diabetic retinopathy**	1.12(0.66–1.91)	0.67		
**Diabetes duration**	0.98(0.94–1.03)	0.41		
**BMI**	1.00(0.93–1.07)	0.99		
**Systolic BP**	1.02(1.01–1.03)	0.00		
**HbA1c**	0.87(0.73–1.02)	0.08		
**Total Cholesterol**	1.17(1.05–1.31)	0.00		
**Alb**	0.94(0.90–0.97)	0.00	0.95(0.90–0.99)	0.01
**Urine protein**	1.15(1.08–1.21)	0.00	1.08(1.01–1.15)	0.03
**Baseline eGFR**	0.96(0.95–0.98)	0.00	0.96(0.95–0.98)	0.00

Clinical Model: Clinical variables with significance at *P* < 0.1 in univariate models (baseline eGFR, proteinuria, albumin, systolic BP, HbA1c, total cholesterol) were included in the multivariable clinical model, and those that remained significant in the multivariable clinical model were baseline eGFR, urine proteinuria and albumin

*Abbreviations*: *Alb* albumin, *BMI* body mass index, *CI* confidence interval, *eGFR* estimated glomerular filtration rate, *ESRD* end-stage renal disease, *HbA1c* glycosylated haemoglobin

**Fig. 5:**
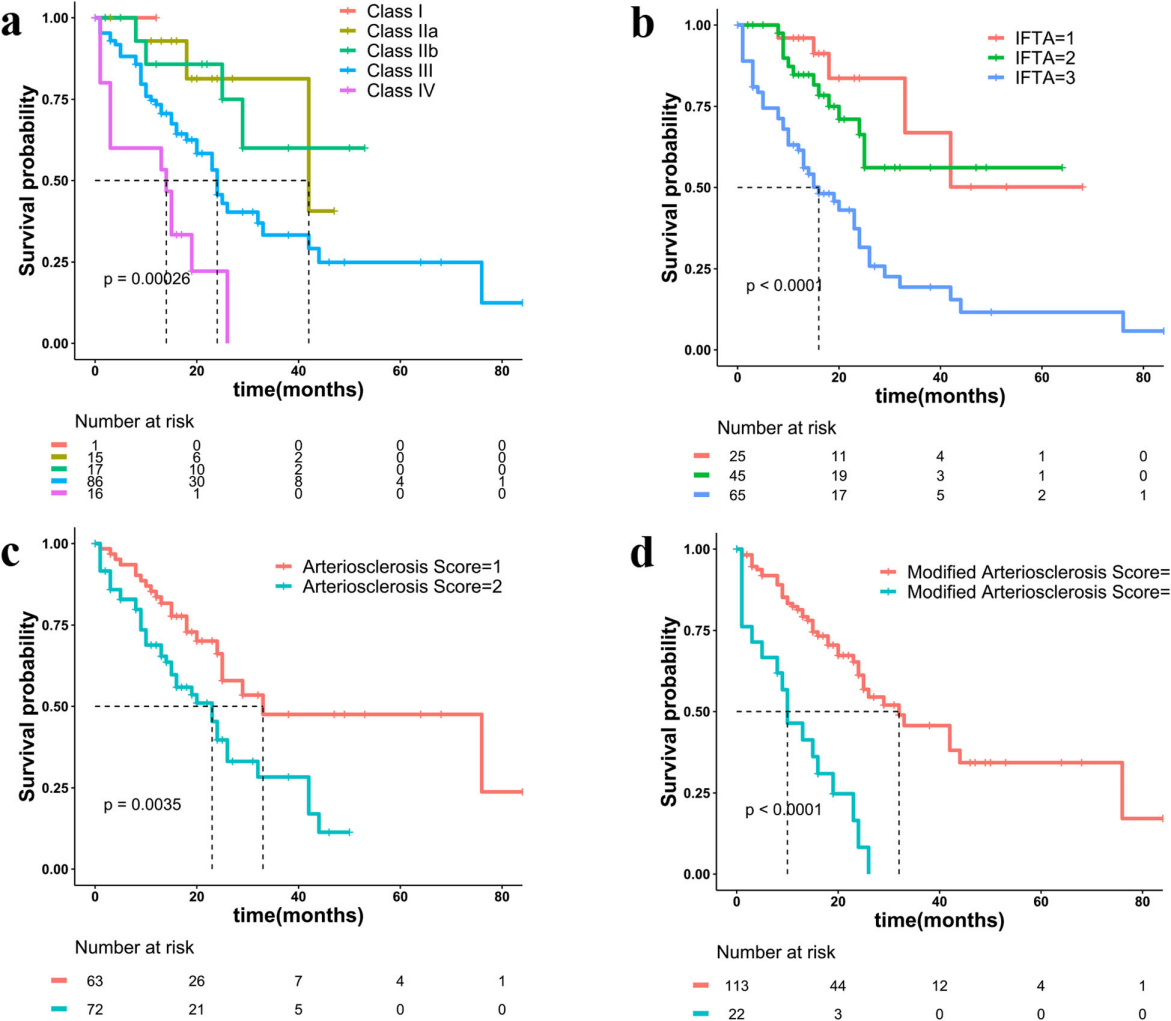
Kaplan-Meier curves of RS according to pathologic variables. **a** Glomerular classification of DN; **b** IFTA; **c** arteriosclerosis score; **d** modified arteriosclerosis score

As shown in Table 4, univariate analysis of pathological factors revealed that RPS glomerular class (hazard ratio, HR: 2.24, 95% confidence interval, CI: 1.47–3.40), IFTA (HR: 2.44, CI: 1.61–3.69), arteriosclerosis score (HR: 2.14, CI: 1.26–3.63) and modified arteriosclerosis score (HR: 4.15, CI: 2.35–7.36) were significantly associated with renal survival. KM survival curve for renal survival was performed. Higher RPS glomerular class (Fig. 5a, *P* = 0.00026), IFTA (Fig. 5b, *P* < 0.0001), arteriosclerosis score (Fig. 5c, *P* = 0.0035) and modified arteriosclerosis score (Fig. 5d, *P* < 0.0001) were correlated with shorter RS time in DKD patients.

**Table 4:** Univariate and multivariable models for time to outcome (endpoint:ESRD or death)

Characteristics	Univariate Model HR (95% CI)	***P*** Value	Pathological Model HR (95% CI)	***P*** Value	Fully Risk-Adjustment Model HR (95% CI)	***P*** Value
**Clinical features**						
**Alb**						
**Baseline Urine protein**					1.10(1.04–1.16)	0.00
**Baseline eGFR**					0.97(0.95–0.98)	0.00
**Pathological features**						
**RPS Glomerular class**	2.24(1.47–3.40)	0.00	1.68(1.13–2.50)	0.01		
**IFTA**	2.44(1.61–3.69)	0.00	1.83(1.18–2.84)	0.01		
**Interstitial inflammation**	1.59(0.96–2.62)	0.07				
**Arteriosclerosis score**	2.14(1.26–3.63)	0.01				
**Modified Arteriosclerosis score**	4.15(2.35–7.36)	0.00	1.44(1.04–1.99)	0.03		
**Arteriolar hyalinosis score**	2.43(0.81–7.34)	0.12				
**Modified Arteriolar hyalinosis score**	1.15(0.79–1.67)	0.46				
**GBM thickness**	1.00(0.99–1.00)	0.86				

Pathological model: RPS, IFTA and Modified arteriosclerosis score; The fully risk-adjustment model: clinical features (eGFR, proteinuria, albumin) and RPS class, IFTA, Modified arteriosclerosis score

*Abbreviations*: *Alb* albumin, *CI* confidence interval, *eGFR* estimated glomerular filtration rate, *ESRD* end-stage renal disease, *GBM* glomerular basement membrane, *IFTA* interstitial fibrosis and tubular atrophy, *RPS* the Renal Pathology Society

**Fig. 6:**
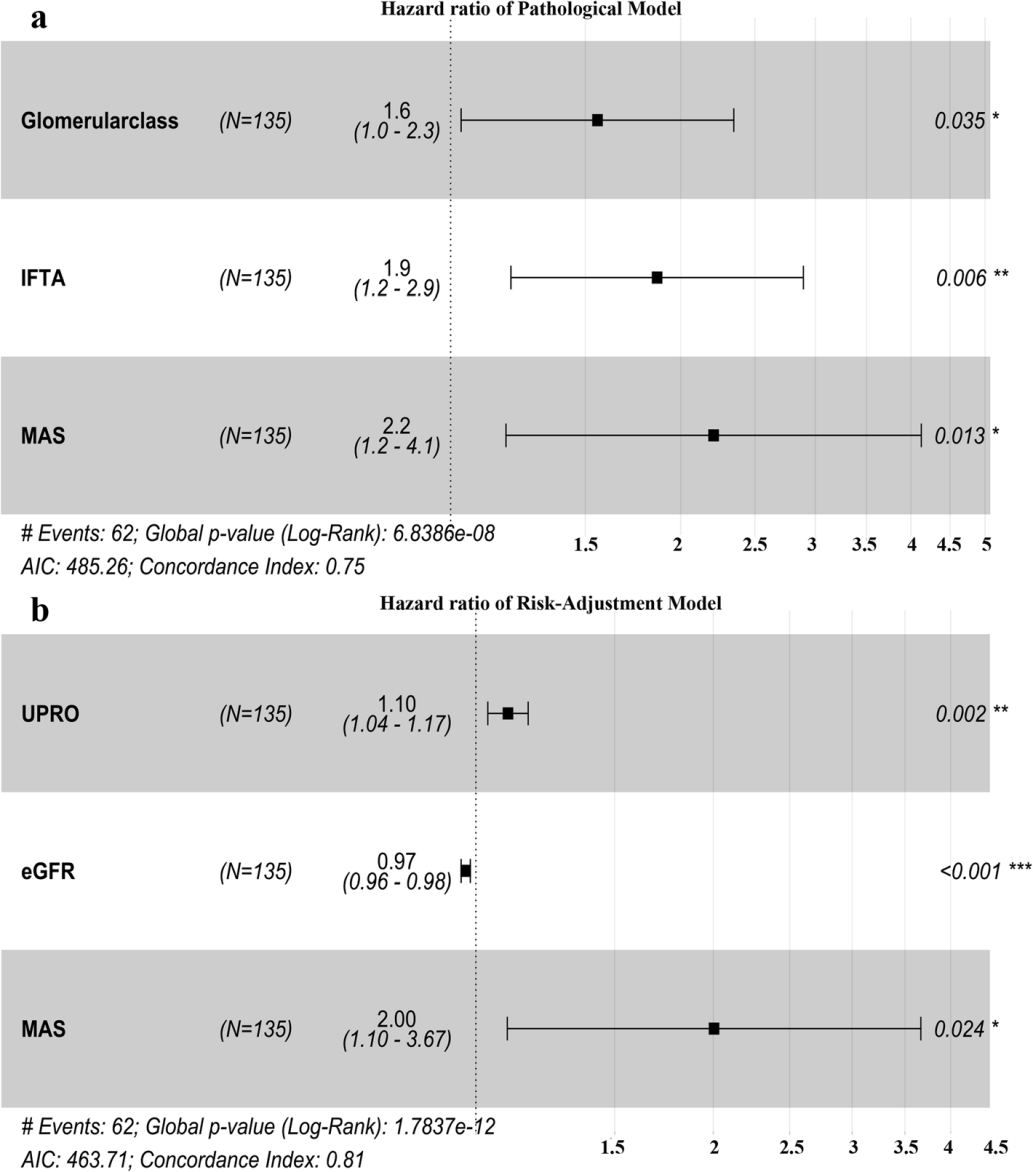
Forest plots of multivariable correction Cox regression analysis for renal survival. **a** Hazard ratio of the pathological model; **b** hazard ratio of the risk-adjustment model. Abbreviations: AIC, Akaike information criterion; eGFR, estimated glomerular filtration rate; IFTA, interstitial fibrosis and tubular atrophy; MAS, modified arteriosclerosis score; UPRO, urine protein

Pathological variables with *P* < 0.05 in univariate analysis were also used to construct two multivariable models. In the pathological model, only RPS glomerular class and IFTA were statistically significant if RPS glomerular class (HR: 1.72, CI: 1.153–2.57), IFTA (HR: 1.96, CI: 1.26–3.06) or arteriosclerosis scores (HR: 1.29, CI: 0.73–2.29) were included. However, if the modified arteriosclerosis score (HR: 2.21, CI: 1.18–4.13) was used instead of the arteriosclerosis score in the model, all three variables were statistically significant. In the second multivariable model, clinical features (baseline eGFR, urine proteinuria and albumin) and RPS glomerular class, IFTA or modified arteriosclerosis score were included. The full risk-adjustment model showed that only baseline eGFR (HR: 0.97, CI: 0.96–0.98), urine proteinuria (HR: 1.10, CI: 1.04–1.17) and modified arteriosclerosis score (HR: 2.01, CI: 1.10–3.67) were independently associated with RS. The forest plot in Fig. 6 shows the results of the stepwise model selection for time to renal survival using multivariable Cox regression.

Figures S1 and S2 show the AUCs of different risk variables and two models (pathological model and full risk-adjustment model) for RS at 24 months after the start of follow-up using time-dependent ROC analysis. In the comparison of individual factors, eGFR showed the highest correlation with RS, with an AUC of 0.81 (95% CI: 0.71–0.92). The full risk-adjustment model had stronger predictive power than the pathological model after correction for multiple factors.

**Fig. 7:**
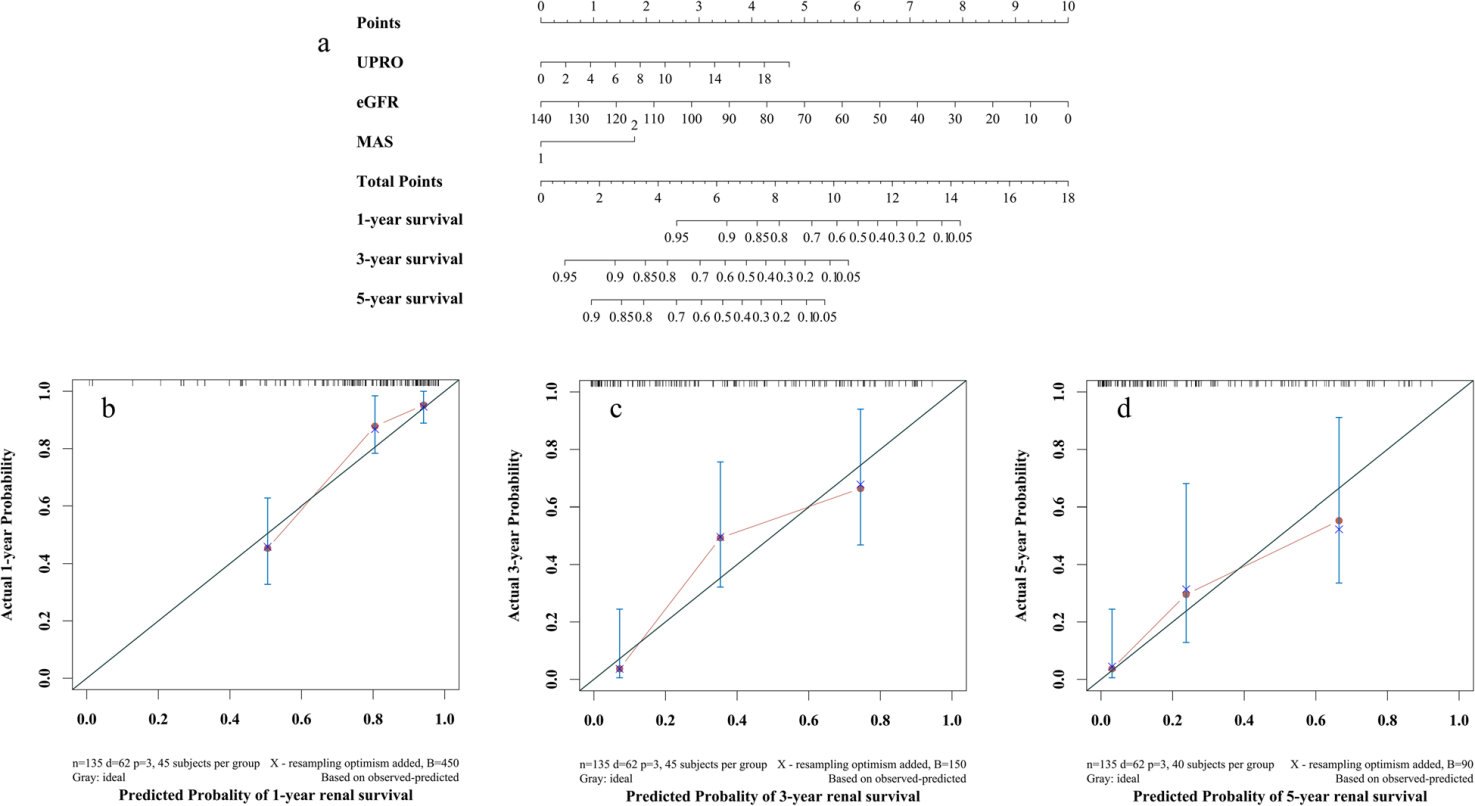
Prognostic nomogram and calibration plots for renal survival. **a** Nomogram to predict the risk of individual renal survival in DKD patients (based on a risk-adjustment model). For each variable contained in the model, points are assigned for each variable by drawing a straight line upward to the “Points” axis. Then, the user can add all these points and locate the number on the “Total Points” axis. The patient’s renal survival was calculated after 1, 3, or 5 years. **b**, **c** and **d** Calibration plots at 1, 3, or 5 years for the final multivariable corrected model. The nomogram-predicted probability of renal survival is plotted on the horizontal axis, and the actual renal survival is plotted on the vertical axis. Abbreviations: UPRO, urinary protein; MAS, modified arteriosclerosis score; eGFR, estimated glomerular filtration rate

A prognostic nomogram for 1-, 3-, and 5-year RS was established (Fig. 7a). Baseline eGFR, urine proteinuria and modified arteriosclerosis score, which were shown to be independent predictors for renal survival in the multivariable Cox regression analysis, were included in the nomogram.

In terms of discrimination, the C-index for renal survival was 0.82 (95% CI: 0.75–0.87). The calibration curve for the probability of survival at 1, 3 or 5 years after biopsy showed optimal agreement between the prediction by the nomogram and the actual observation (Fig. 7b-d). According to the calibration curve, the predicted survival probabilities at 1, 3 and 5 years were in line with the actual survival probability, and the confidence interval intersected with the 45° diagonal. In addition, the confidence interval of some prediction probabilities was very wide, which indicated that the sample size needs to be increased for further assessment.

## Discussion

Herein is a detailed study of the association of advanced vascular lesions and clinical outcomes in patients with biopsy-proven DKD. With respect to the first research question, we found that the proportion of severe arteriosclerosis was an independent predictor of clinical progression to ESRD for DKD patients. In lupus nephritis and IgA nephropathy, some studies have supported vascular lesions as independent risk factors for predicting renal disease progression [32–35]. Although the pathological classification of DKD included the vascular score in the original literature, the authors did not validate the relationship between vascular lesions and renal prognosis [12]. Furthermore, we found that studies implementing specific analyses based on the correlation of renal vascular lesions and renal prognosis were scarce.

Yu An et al. [36] studied more than 300 patients with type 2 DKD confirmed by biopsy and found that vascular lesion scores demonstrated no association with renal outcomes. However, a different study demonstrated that the presence of arteriolar hyalinosis and arteriosclerosis were associated with renal replacement therapy (RRT) initiation in univariate analyses, although there was no statistical significance in multivariable analyses [37]. The original classification of arteriosclerosis in DKD only assessed the most severe arteriosclerosis but did not assess the proportion of the arteries involved. This may have caused sampling error, and we therefore revised the new scoring systems for vascular lesions to highlight the extent of involvement throughout the tissue. In our study, we found that the higher the proportion of severe arteriosclerosis was, the poorer the baseline renal function, and the more rapidly the creatinine level progressed in the future. Similar to previous research on lupus nephritis, we also found that the proportion of arteriosclerosis was associated with proteinuria. Therefore, we established a new arteriosclerosis score based on the proportion of severe arteriosclerosis.

In the present study, the original arteriosclerosis score was a risk factor in univariate analysis but not in multivariable analysis (Fig. 5c, Table 4). However, univariate and multivariable analyses demonstrated that the modified arteriosclerosis score was an independent factor for prognosis in DKD (Fig. 5d, Fig. 6, and Table 4). Compared with the previous scoring system, we believe the new scoring system can distinguish DKD, which is more likely to worsen. Bohle et al. [38] described that increases in vascular disease correlated with more severe glomerular disease. Gambara et al. [39] also described different renal patterns in diabetic patients that led to renal dysfunction, a subset of which was due to significant vascular sclerosis. Salvatore et al. [40] proposed that the spectrum of ischaemic glomerular disease and podocyte injury may depend on the degree and duration of micro- and macrovascular occlusion and that severe vascular changes in diabetic nephropathy lead to hypoxic injury.

Arteriolar hyalinosis is another indicator of vascular lesions. We also modified this scoring system; however, neither the original indicator nor the improved indicator was statistically significant in our study. This result is controversial, however, because some studies have suggested that, similar to the role of hyaline arteriolosclerosis in renal transplantation [41, 42], this pathological feature is associated with the progression of diabetic kidney disease [40]. This phenomenon might be associated with the prevalence of severe arteriolar hyalinosis in our DKD cohort. More than one vessel of moderate to severe arteriolar hyalinosis was commonly observed in 88.9% of patients with diabetic nephropathy, suggesting that this index in the classification was incapable of discriminating lesions with various severities for our cohort. This result was similar to a previous study [36].

Glomerular lesions are the most characteristic pathologic changes of DKD. Although the glomerular classification of RPS aims to help reveal the progression of DKD, its prognostic value remains to be verified. In their recent study on patients with biopsy confirmed DKD, Mottl et al. [14] found that glomerular pathologic classification had statistical significance for predicting renal prognosis only in the single-factor analysis; however, it was not statistically significant in the multifactor analysis. Two other research teams also reported that the renal prognostic significance of the glomerular pathologic classification lost power after multivariate correction [16, 43]. In contrast, several studies have shown a significant correlation between the RPS DKD classification and decreased GFR after multivariate correction [36, 44–46]. With regard to tubulointerstitial lesions, studies conducted by An Yu et al. [36] and Mise et al. [46] suggested that the IFTA score is an independent risk factor for renal prognosis of diabetic nephropathy, but the research conducted by Mottl et al. [14] suggested that IFTA has no statistical significance for renal prognosis of DKD. In our study, glomerular lesions and IFTA were statistically significant in both univariate and pathological models but not after adjustment for proteinuria, eGFR, serum albumin levels, or arteriosclerosis.

On the basis of previous work, we established a novel user-friendly renal survival prediction model built on three key parameters (urine protein, eGFR and modified arteriosclerosis score) and provided a prognostic nomogram and score [20]. As a tool to predict clinical prognosis, nomograms are widely used in oncology and other medical aspects to help clinical decision-making. Nomograms satisfied our need for an integrated biological and clinical model to realize the goal of personalized medicine; thus, it is reasonable to predict renal prognosis of DKD with a nomogram. The present work revealed considerable heterogeneity among DKD patients regarding their renal survival risk profiles. Our nomogram could provide the nephrologist with a precise probability of renal survival in patients with DKD and might offer an opportunity to define risk-adapted strategies for DKD management in the future.

Our study has multiple strengths. The main strength of our study is that we illustrated the modified arteriosclerosis score vascular lesion as an independent marker of time to diabetic ESRD with a modified definition. Our method could identify DKD patients with a worse prognosis. Furthermore, the new immunohistochemical method we performed showed more comprehensive characteristics of vascular lesions than conventional staining, which may be used as a reference for the pathological score of DKD in the future. Third, our study was constructed in the framework of a multicentre cohort study with a broad spectrum of parameters available at diagnosis. Multicentre studies can better represent real-world heterogeneity and are more generalizable than single-centre studies. Moreover, we created a nomogram for DKD patients to help nephrologists make clinical decisions. Nomograms have the ability to generate an individual probability of a clinical event by integrating diverse prognostic and determinant variables, which meets our desire for biologically and clinically integrated models and fulfils our drive towards personalized medicine. Finally, the blinded evaluation of renal biopsy is another strength of this study.

Despite these strengths, some limitations must be considered when interpreting our findings. First, it was a retrospective study. However, all end points were based on objectively measured laboratory values that reduced the chance for bias. Second, it was a small sample size. Therefore, our findings are hypothetical and need to be reexamined with a larger data set. Third, we did not evaluate the therapeutic interventions during follow-up, which may have had miscellaneous impacts on renal prognosis. Finally, histologic changes were assessed based on a simple classification, but diabetic glomerulopathy is far more complicated.

In conclusion, we present here a novel renal survival prediction model based on three key independent prognostic factors. Other studies are required to determine whether the modified arteriosclerosis score can be applied as a novel, independent marker of time to ESRD for DKD patients. The prognostic nomogram and the score proposed may help offer the opportunity to define risk-adapted strategies for DKD management in the future.

## Supplementary Information

**Additional file 1: Figure S1.** Time-dependent ROC of different risk factors for RS at 24 months of follow-up. Abbreviations: AUC, area under curve; CI, confidence interval; eGFR, estimated glomerular filtration rate; IFTA, interstitial fibrosis and tubular atrophy; MAS, modified arteriosclerosis score; UPRO, urine protein.

**Additional file 2: Figure S2.** Time-dependent ROC of different models for RS at 24 months of follow-up.

## Data Availability

The datasets created and/or analysed during the current study will be available from the corresponding author on reasonable request. There are no security, licensing, or ethical issues related to these data.
