# Supplementary figures and images for: Modified arteriosclerosis score predicts the outcomes of diabetic kidney disease
Source: BMC Nephrol. 2021 Aug 18;22:281. doi: 10.1186/s12882-021-02492-x (PMC8375127; doi:10.1186/s12882-021-02492-x)

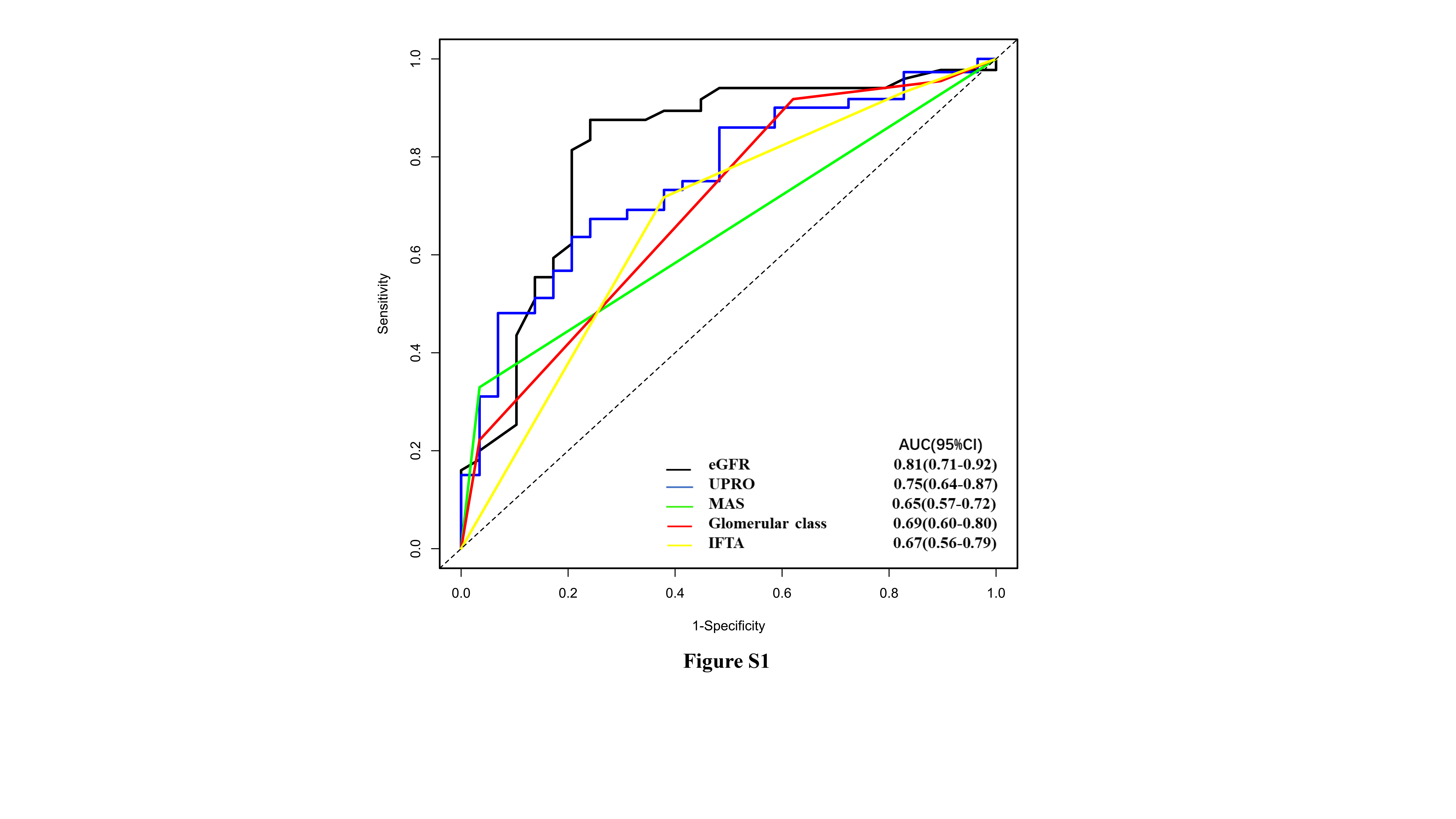

Supplement: Supplementary file 1 — Additional file 1: Figure S1. Time-dependent ROC of different risk factors for RS at 24 months of follow-up. Abbreviations: AUC, area under curve; CI, confidence interval; eGFR, estimated glomerular filtration rate; IFTA, interstitial fibrosis and tubular atrophy; MAS, modified arteriosclerosis score; UPRO, urine protein. [file 12882_2021_2492_MOESM1_ESM.tif]

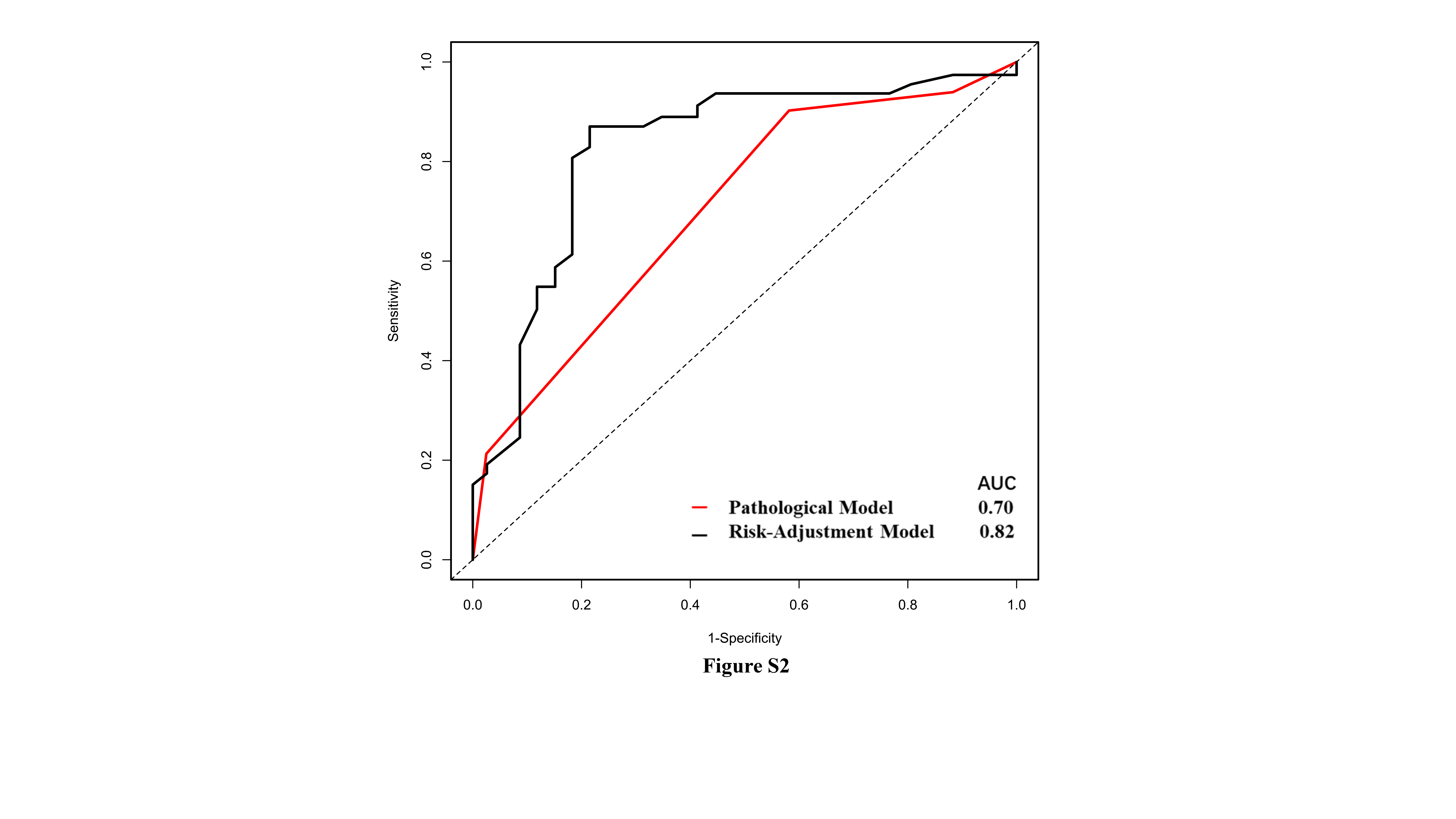

Supplement: Supplementary file 2 — Additional file 2: Figure S2. Time-dependent ROC of different models for RS at 24 months of follow-up. [file 12882_2021_2492_MOESM2_ESM.tif]
